# Investigations on Transfer of Pathogens between Foster Cows and Calves during the Suckling Period

**DOI:** 10.3390/ani11092738

**Published:** 2021-09-19

**Authors:** Katharina Köllmann, Nicole Wente, Yanchao Zhang, Volker Krömker

**Affiliations:** 1 Department of Microbiology, Faculty of Mechanical and Bioprocess Engineering, University of Applied Sciences and Arts, 30453 Hannover, Germany; katharina.marie.koellmann@tiho-hannover.de (K.K.); nicole.wente@hs-hannover.de (N.W.); yanchao.zhang@hs-hannover.de (Y.Z.); 2 Department of Veterinary and Animal Sciences, University of Copenhagen, 1870 Frederiksberg, Denmark

**Keywords:** foster cows, calf rearing, suckling, udder health, *Pasteurella multocida*

## Abstract

**Simple Summary:**

Regarding the increasing importance of alternative calf rearing systems, such as the foster cow system, better knowledge about the transfer of pathogens via suckling calves would be very helpful. In this context, the present study was conducted on a large organic dairy farm practising calf rearing on foster cows. For microbiological examinations, quarter milk samples were collected both from foster cows and biological dams. Additionally, swabs were taken from the oral cavities of the associated foster calves. The concordance of pathogens between cows and calves was further examined by strain comparisons. The same strains of isolates were detected for *Pasteurella multocida*, *Staphylococcus aureus*, *S. sciuri* and *Streptococcus (Sc.) suis*. Based on the present results and the available literature, transmission of *P. multocida* and *S. aureus* via suckling calves is considered very likely. Transmission of pathogens from the dam to the foster cow with the suckling calf as a vector could not be detected.

**Abstract:**

To date, there have been few studies on the health effects of foster cow systems, including the transmission of mastitis-associated pathogens during suckling. The present study aimed to compare the pathogens detected in the mammary glands of the foster cow with those in the oral cavities of the associated foster calves and to evaluate the resulting consequences for udder health, calf health and internal biosecurity. Quarter milk sampling of 99 foster cows from an organic dairy farm was conducted twice during the foster period. Oral cavity swabs were taken from 345 foster calves. Furthermore, quarter milk samples were collected from 124 biological dams to investigate possible transmission to the foster cows via the suckling calves. All samples were microbiologically examined and confirmed by MALDI-TOF (matrix-assisted laser desorption time-of-flight mass-spectrometry). Using RAPD-PCR (randomly amplified polymorphic DNA polymerase chain reaction), strain similarities were detected for *Pasteurella multocida, Staphylococcus aureus*, *S. sciuri* and *Streptococcus (Sc.) suis*. Transmission of *P. multocida* and *S. aureus* probably occurred during suckling. For *S. sciuri* and *Sc. suis*, environmental origins were assumed. Transmission from dam to foster cow with the suckling calf as vector could not be clearly demonstrated.

## 1. Introduction

Growing public interest in animal welfare in recent years has been accompanied by an increasing number of people opposing the early separation of dairy cows and calves [1,2,3]. As a result, cow–calf contact systems with physical contact between cows and calves over longer periods of time are coming more into focus [2,4,5]. The foster cow system is one possible way to implement such an alternative rearing system. Two to four calves are reared by a foster cow, which is usually not milked. The foster cow‘s own calf may also be among the calves [6]. Thus, the foster cow system is considered a workable compromise between early separation and more natural calf rearing since the calves are raised by a foster cow rather than their biological dam [7]. At the same time, the high milk yielding potential of the modern dairy cow is used to rear multiple calves [8].

So far, the available literature on the effects of such a rearing system on the health of foster cows and calves is scarce. With regard to the foster cow, some studies investigated the influence of multiple suckling on udder health. Walsh [9] determined a reduced incidence of intramammary infections (IMI) in 12 cows nursing four calves twice daily compared to 12 machine milked cows in the first 100 days of lactation. In the foster cows, 2.9% of the quarters showed an IMI, as opposed to 29.9% of the quarters in non-suckled cows. Margerison et al. [10] examined cows of a tropical breed which were either suckling their own calves, had multiple foster calves or were exclusively milked. Suckling was associated with a significant reduction in somatic cell count (SCC), and the effect was more pronounced when multiple calves were suckled (own calf: 95,000 cells/mL, foster calves: 85,000 cells/mL, milked: 106,000 cells/mL). The improvement in udder health was mainly attributed to the more effective udder emptying by the calves [10,11]. Other authors could not detect significant differences in SCC or the incidence of IMI between foster cows and machine milked cows [7,12,13]. The comparability of these studies is limited due to different study designs, but suckling multiple calves seems to have a tendency for positive effects on udder health. None of the known studies showed a significant negative effect of suckling, although publication bias cannot completely be excluded. Nevertheless, some authors are concerned that permanent suckling leads to incomplete closure of the teat. This could facilitate the entry of pathogens into the mammary glands [7].

Furthermore, there is a lack of studies investigating the extent of pathogen transfer between cow and calf during suckling. In some older studies, pathogens detectable in quarter milk samples were also found in the oral cavities of calves. For instance, Klein and Kleckner [14] examined the transmission of *Streptococcus (Sc.) agalactiae* by suckling calves. The calves suckled twice daily first from infected and then from non-infected cows. Oral swabs were taken from four calves at various times after suckling, and *Sc. agalactiae* was regularly detected up to eight hours thereafter. Schalm [15] examined the prevalence of *Sc. agalactiae* in the oral cavities of four calves after ingesting milk from infected cows. The calves cross-suckled the teats of their pen mates and in one of them the pathogen was subsequently isolated from the teats. Persistence in the mammary glands until the first calving was suspected, as one heifer excreted *Sc. agalactiae* with her milk. In contrast, Rigby et al. [11] could not demonstrate *Staphylococcus aureus* in saliva samples of calves after suckling infected cows. The same applies to *Pasteurella multocida*, which was not detectable in nasal and oral cavity swabs of three suckling calves after a herd outbreak. However, transmission via suckling calves is considered a possible route of infection [16].

Regarding the foster cow system, it should also be taken into account that many calves suckle on more than one cow, especially if their dam is not in the group [17]. Kilgour [18] mentioned the risk of pathogen transmission between different cows by alternating suckling calves. In a natural environment, this is considered unlikely since calves usually exclusively suckle their dam [18].

However, most of these studies are relatively old, not methodically designed, involve few experimental animals and have different objectives. Regarding the growing importance of alternative calf rearing systems, further studies are required. Therefore, the present study was conducted as a pilot study. The objective was to detect transmission of pathogens between cows and calves during suckling. For this purpose, the prevalence of mastitis-associated pathogens in the oral cavities of foster calves at the end of the suckling period was investigated and the pathogens detected were compared with those in the mammary glands of the associated foster cow. In addition, the present study aimed to examine whether the mastitis-associated pathogens of the dam were also found in the udder of the foster cow at a later point in time. The consequences for udder health, calf health and internal biosecurity were evaluated.

## 2. Materials and Methods

### 2.1. Farm and Management

The study was conducted between May and November 2020 on a large organic dairy farm in Eastern Germany with an average of 1500 milking Holstein Friesian cows producing an average of 7908 kg milk per year (ECM). Cows were milked twice daily in a rotary parlour and kept in a cubicle housing system with access to pasture between April and October. The farm was selected due to its special calf rearing method of using foster cows and the occurrence of all relevant mastitis pathogens in its herd, including contagious pathogens such as *S. aureus* or *Sc. agalactiae*.

After calving, cows and calves were left together in a group with up to four other newborn calves and their dams. During this time, the cows were milked twice daily on a pipeline milking system and observed for good foster cow characteristics, like allowing other calves to suckle or a convenient udder conformation. Cows that had given birth to twins were preferably used as foster cows. After 3–5 days, the calves were assigned to the foster cows, while the remaining dams returned to the milking herd. Usually three calves, occasionally four, were reared by one foster cow. Before introducing the foster cow and her calves to a larger group of usually 15 other foster cows and their calves, they were given another week in small groups with two other foster cows and their associated calves to get used to each other.

Foster cows and calves were kept separate from the milking herd in an open barn with slatted flooring with a resting area made of straw and a separate area with permanent access for calves only. All foster cows were checked daily by the staff for any signs of clinical mastitis such as reddening, swelling, pain, increased heat of the udder or abnormal milk appearance. If treatment was required, the foster cows were removed from the group. The suckling period ended after about 3.5 months. Prior to that, the foster calves were gradually weaned. Subsequently, the foster cows returned to the milking herd and were milked twice daily until the following dry period.

### 2.2. Study Design and Data Collection

Initially, a total of 124 primiparous and multiparous foster cows were included in the study, which all calved between May and August 2020. Lactation numbers varied from one to eight, with the majority of foster cows being in their second to fourth lactation (99 cows) and twelve being heifers. Quarter milk samples from all four quarters were collected from 99 of these foster cows for microbiological diagnosis and SCC at the beginning and end of the suckling period (first (F1) and second (F2) sampling of foster cows). There were no selection criteria except calving in the mentioned period. Furthermore, quarter milk samples of 124 primiparous (32) and multiparous cows, which were the biological dams of some of the foster calves, were taken 3–12 days after calving within the first week after integration in the milking herd (sampling of dams (D1)). These samples were also subjected to microbiological diagnosis and SCC.

At the end of the suckling period, dry swabs were taken from the oral cavities of 345 foster calves (sampling of calves (C1)). This sampling was conducted simultaneously to the F2 sampling of the associated foster cows. 

**Figure 1:**
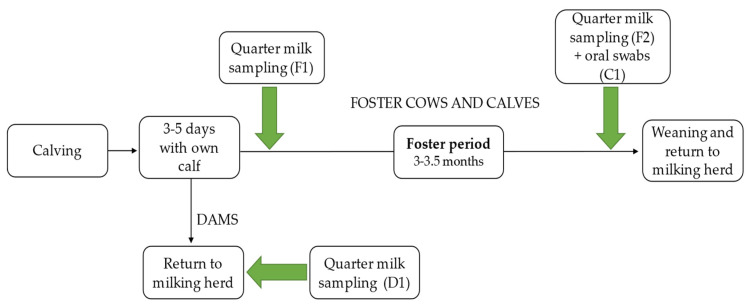
Scheme of sampling. F1 = first quarter milk sampling of foster cows. F2 = second quarter milk sampling of foster cows. C1 = oral cavity swabs of foster calves. D1 = quarter milk sampling of dams.

The scheme of sampling is shown in Figure 1.

### 2.3. Quarter Milk Samples

The quarter milk samples of dams and foster cows were aseptically collected in sterile plastic tubes by the veterinarian and the farmer. The test tubes contained boric acid as a preserving agent. Before each collection, the first milk squirts were discarded and the apex of the teat was cleaned and disinfected with 70% ethanol. Disposable gloves were worn during sampling and disinfected after each cow. The collected milk samples were either directly transported in a cooling box or sent by mail to the laboratory (Department of Microbiology, Faculty of Mechanical and Bioprocess Engineering, University of Applied Sciences and Arts Hannover, Germany) for further analysis.

### 2.4. Oral Cavity Swabs

To collect the oral swabs, the calves were restrained by a feeding fence. A helper opened the calves’ mouth using a wooden mouth wedge with an opening in the middle through which the veterinarian took the sample with a sterile dry swab. Taking into account the defensive movements of the calves, the swab was taken from the buccal cavity or as far back in the throat as possible from the tonsils. Calves that had just eaten were sampled later to avoid contamination by feed residues. In between, the mouth wedge was washed in iodine soap containing povidone iod (Novasan Plus Jodseife, Ferd. Eimermacher GmbH and Co. KG, Nordwalde, Germany). After sampling, the swabs were transported in sterile test tubes containing 2 mL brain-heart-broth (Merck KGaA, Darmstadt, Germany) and shipped to the departmental laboratory in a cooling box for further analysis at the day of sampling.

### 2.5. Laboratory Analyses

#### 2.5.1. Quarter Milk Samples

The examination of the quarter milk samples was conducted in the laboratory following the guidelines of the German Veterinary Medical Association [19].

First of all, 10 µL of each sample were plated on esculin blood agar (5% defibrinated sheep blood, Oxoid Deutschland GmbH, Wesel, Germany) in duplicate and aerobically incubated at 37 °C. The blood agar plates were analysed after 24 and 48 hours. A first classification was based on Gram-straining, colony and cell morphology, haemolysis and esculin hydrolysis. Further differentiation was carried out using various tests. The clumping factor test (Staph Plus Latex Kit, DiaMondiaL, Virotech Diagnostics GmbH, Vienna, Austria) was used for Gram-positive, catalase-positive and ß-haemolysing cocci to identify *S. aureus* colonies. Gram-positive and catalase-positive cocci without ß-haemolysis were categorised as non-*aureus* staphylococci (NaS). Serological tests (Strep Latex Kit, DiaMondiaL, Virotech Diagnostics GmbH, Vienna, Austria) were used for Gram-positive, catalase-negative and esculin non-hydrolysing cocci to distinguish between Lancefield Group B (*Sc. agalactiae*) and C (*Sc. dysgalactiae*). Gram-positive, catalase-negative and esculin-hydrolysing cocci were plated on modified Rambach agar to check for ß-D-galactosidase activity [20]. A positive reaction became visible as a blue colour change and was designated as *Sc. uberis*. On the contrary, ß-D-galactosidase-negative cocci were identified as enterococci. Gram-positive curved and catalase-positive bacilli were classified as *Corynebacterium* ssp., whereas Gram-positive curved but catalase-negative and ß-haemolysing bacilli were identified as *Trueperella (T.) pyogenes*. Yeasts were categorised according to their cell morphology. Gram-negative bacilli were differentiated by using the Oxidase Test (Bactident Oxidase, Merck KGaA, Darmstadt, Germany) to prove the presence of cytochromoxidase C and the oxidation/fermentation test (glucose supplemented oxidation-fermentation test medium, Merck KGaA, Darmstadt, Germany) to detect the oxidation or fermentation of carbohydrates as well as to test the microorganisms for mobility. Oxidase negative, glucose-metabolizing colonies were further plated and incubated on Chromocult^®^ Coliform agar (Merck KGaA, Darmstadt, Germany) to distinguish between *Escherichia coli* and other coliforms. Non-motile coliforms were assigned to *Klebsiella* spp. Cytochromoxidase positive bacilli, which metabolised glucose oxidatively, were classified as *Pseudomonas* spp. On the other hand, cytochromoxidase positive bacilli with a fermentative glucose degradation are referred to as *Pasteurella* spp. If more than two different colony types were found on one blood agar plate, the milk sample was assumed to be contaminated, except for when a colony of a contagious microorganism (*S. aureus, Sc. agalactiae*, *Sc. dysgalactie* or *T. pyogenes*) was found.

SomaScope^TM^ Smart (Delta Instruments B.V., Drachten, The Netherlands) was used to determine the SCC of the milk samples.

#### 2.5.2. Oral Cavity Swabs

The oral swabs of the calves were first vortexed (Vortex Genie2, Scientific Industries Inc., Bohemia, NY, USA) for approximately 30 s. Ringer’s solution was used to prepare the dilution series 10^−1^, 10^−2^ and occasionally 10^−3^ if a contamination of the swab was obvious. A total of 100 µL of each sample were spread over the entire surface of an esculin blood agar plate (5% defibrinated sheep blood, Oxoid, Deutschland GmbH, Wesel, Germany) using a Drigalski spatula. Subsequently, the blood agar plates were aerobically incubated at 37 °C for 48 h. The following bacteriological examination focused on pathogens present in the quarter milk samples of the dam and the associated foster cow, and typical mastitis-associated pathogens, since most plates showed growth of a variety of different pathogens. For example, if NaS were identified in one of the quarter milk samples of the foster cows, the oral swabs were checked for Gram-positive and catalase-positive cocci. Up to seven isolates per swab were collected from these colonies for further diagnostics.

The bacteriological results of both the quarter milk samples and the oral swabs were confirmed by Matrix-assisted laser desorption time-of-flight mass-spectrometry (MALDI-TOF, Bruker Daltonics GmbH & Co. KG, Bremen, Germany) with a direct transfer method without acid extraction, using the MBT Compass Library (Revision F, MBT 84,668 MSP Library, Bruker Daltonics GmbH & Co. KG, Bremen, Germany). The cut-off level was ≥1.7 for species identification [21]. All isolates were stored at −80 °C with 800 µL of brain-heart broth (Merck KGaA, Darmstadt, Germany) and 200 µL of glycerol until further analysis.

#### 2.5.3. Strain Comparisons

Quarter milk samples and oral swabs in which the same pathogen was detected in the calf and the associated foster cow or dam, based on the results of the MALDI TOF, were further investigated for strain diversity by randomly amplified polymorphic DNA-polymerase chain reaction (RAPD-PCR; [22]). The extraction of the bacterial DNA was conducted using the DNeasy Blood and Tissue Kit (Qiagen Benelux B.V., Venlo, The Netherlands) in accordance with the manufacturer’s instructions. The subsequent RAPD-PCR was carried out in Mx3005 P qPCR system (Agilent Technologies, Inc., Santa Clara, CA, USA) with a reaction mix containing 12.5 µL of ReadyMix^TM^ Taq PCR Reaction Mix (Sigma-Aldrich Chemie GmbH, Taufkirchen, Germany), 5 µL DNA template, 10 pmol of each primer and distilled deionised water to reach the final volume of 25 µL. The following primers were used: ERIC-1R (5′-ATGTAAGCTCCTGGGGATTCAC-3′) [23], OPE-04 (5′-GTGACATGCC-3′) [24], C-RAPD-Staph (5′-CGGGGGACTGTTGGGCGCCATCT-3′) [25] and Primer A (5′-CTGGCGGCTTG-3′) [26]. Further details are given in Table S1 of the supplementary material. After RAPD-PCR, each reaction batch was stained with 2 µL of Midori Green Direct (Nippon Genetics Europe GmbH, Düren, Germany) and separated by electrophoresis on a 2% agarose gel for 1.5 h at 150 V. Either the 250 bp or 100 bp DNA ladder (Carl Roth GmbH + Co.KG, Karlsruhe, Germany) was run in each gel as the molecular size marker. The gels were subsequently visualised using the software GeneSnap (Syngene International Ltd, Cambridge, UK). Identical banding patterns were defined as the same strain.

### 2.6. Statistical Analyses

Data were collected in Microsoft Excel 2016 (Microsoft Corporation, Redmond, WA, USA). The program SPSS (26.0, IBM Corp., Armonk, NY, USA) was used for univariable statistical analysis of the quarter milk samples taken from the foster cows. Each udder quarter was considered as a statistical unit. The χ^2^ –test (Chi-Square-Test) was used to test the differences in pathogen distribution between the two examinations. Statistical significance was given at a *p*-value < 0.05.

## 3. Results

### 3.1. Numbers of Cows, Udder Quarters and Calves

At the beginning, a total of 124 Holstein Friesian foster cows with 486 lactating udder quarters participated in the first examination shortly after calving. Of these cows, 25 left the foster cow–calf group before the final examination due to illness (mainly mastitis) or death. In addition, since no milk could be obtained from eleven quarters during one of the examinations, a total of 385 lactating quarters from 99 cows were finally sampled twice.

The quarter milk samples of the biological dams were collected as a sample of 124 cows with 483 lactating udder quarters. These dams were not used as foster cows.

Oral cavity swabs of the associated foster calves were taken shortly before weaning at an age of approximately 3 to 3.5 months. This sampling finally included 345 foster calves, as some were not able to be captured or had died. 

### 3.2. Quarter Milk Samples

The detailed pathogen distribution of the examined quarter milk samples is shown in Table 1. For the 99 remaining foster cows the bacteriological results of the examination (F1) at the beginning of the foster period and of the second examination (F2) shortly before weaning the calves are presented. 

In F1, mastitis-associated pathogens were detected in 31.4% (*n* = 121) of the quarters. A total of 62.9% (*n* = 242) showed no bacterial growth on blood agar and 5.7% (*n* = 22) were contaminated. At the end of the suckling period, the percentage of quarters with an IMI amounted to 31.7% (*n* = 122). In 58.4% (*n* = 225) of the samples, no bacterial growth was detectable, and 9.9% (*n* = 38) were contaminated. Thus, 7% fewer quarters showed no bacterial growth at the end of the suckling period compared to the first days after calving. The distribution of pathogens was significantly different between the two examination times (*p* < 0.001).

Mastitis-associated pathogens were found in 30.4% (*n* = 147) of the quarter milk samples taken from the dams, whereas in 53.8% (*n* = 260), no bacterial growth was detected and 15.7% (*n* = 76) were contaminated. For both the foster cows and the dams, opportunistic pathogens such as NaS and *Cornyebacterium* spp. constituted the most frequently detected pathogen group. *Pasteurella* spp. (*n* = 13, 3.4%) were found in high percentages in the second examination of the foster cows. In contrast, no single case was detected in the dams. Cow-associated pathogens such as *S. aureus*, *Sc. dysgalactiae* and *T. pyogenes* were involved in a small number of quarters with bacterial growth in both groups.

### 3.3. Oral Cavity Swabs

In Table 2, the results of isolates taken from the oral swabs of the calves after examination by MALDI TOF are presented.

A total of 1548 isolates were identified at species level, and in 695 cases, identification proved impossible. NaS constituted the most frequently detected pathogen group with 34.3% (*n* = 531). Among these, *S. xylosus* (*n* = 195) and *S. haemolyticus* (*n* = 107) were the most common species. Streptococci were the second most frequently identified pathogen group and accounted for 27% (*n* = 418) of all identified isolates. *Sc. pluranimalium* represented the largest proportion with a total of 271 identifications. Mastitis-associated pathogens were detected to a small extent, including *S. aureus* (*n* = 41) and *T. pyogenes* (*n* = 10). *Sc. agalactiae* and *Sc. uberis* were not detected.

### 3.4. Pathogen Matches between Foster Cows and Calves

**Figure 2:**
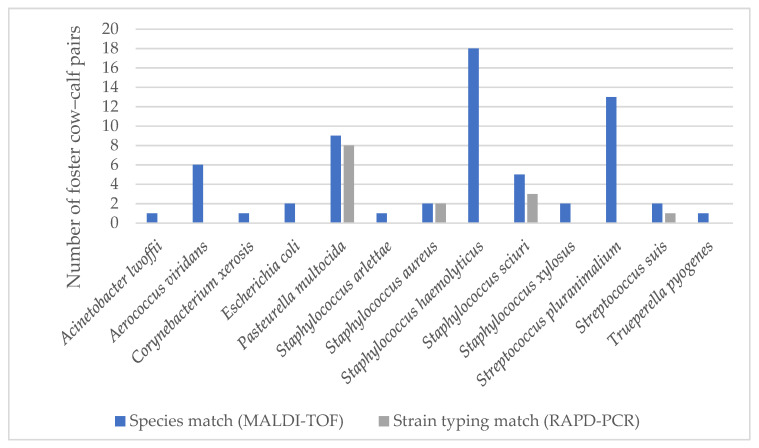
Matched pathogens between foster cows and associated calves after examination with MALDI-TOF and RAPD-PCR.

Figure 2 shows the matched pathogens between foster cows and calves after examination with MALDI-TOF (species match) and RAPD-PCR (strain typing match) as a bar chart. The corresponding table can be found as Table S2 in the supplementary material.

The number of foster cow–calf pairs in which the same pathogen was found both in the quarter milk sample of the foster cow and the oral swab of the associated foster calf is indicated. The results of the MALDI-TOF are shown in blue and those of the RAPD-PCR in grey. For instance, *Acinetobacter lwoffii* was detectable in the quarter milk sample of a foster cow and in the oral cavity of one of her associated calves. Based on chromosome banding, the two isolates showed no match in the subsequent RAPD-PCR. Matches of banding patterns were detected for *P. multocida*, *S. aureus*, *S. sciuri* and *Sc. suis*. Of these, *P. multocida* showed the most matches and two different strains were identified (strains A and B). Strain diversity was considered in the quarters of six foster cows and oral swabs of nine associated calves. Since the isolate of one oral swab could not be cultured after storing, eight isolates remained. All of them showed matching strains between the foster cows and their associated calves. In addition, strain B was also detectable in the quarters of two foster cows from another group.

For *S. aureus*, all isolates with species matches were taken from one foster cow and two of her associated calves and from two other foster cows of the same group. According to RAPD-PCR, the same strain occurred in all samples.

**Figure 3:**
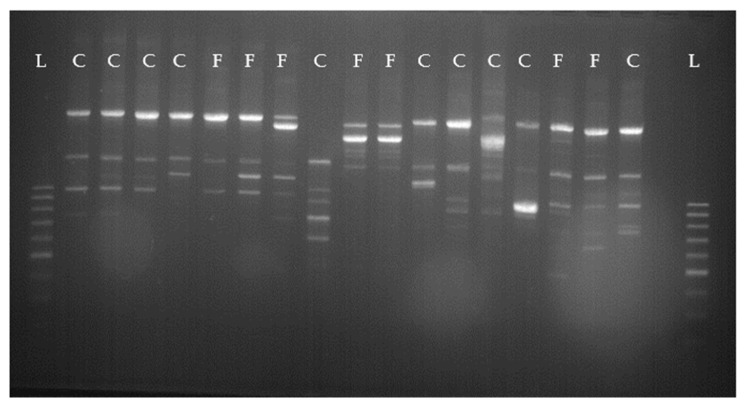
*Staphylococcus haemolyticus*-RAPD-PCR types from different foster cows and calves. L = 100 bp ladder; C = calf; F = foster cow.

NaS constituted the most prevalent pathogen group identified in both quarter milk samples and oral cavity swabs. Of these, *S. haemolyticus* and *S. xylosus* were the most frequently detected pathogen species in the oral cavities of the calves. However, they showed high strain diversity and no strain similarities with those detected in the quarter milk samples of the corresponding foster cows. An overview of some *S. haemolyticus* strains is exemplified in Figure 3.

**Figure 4:**
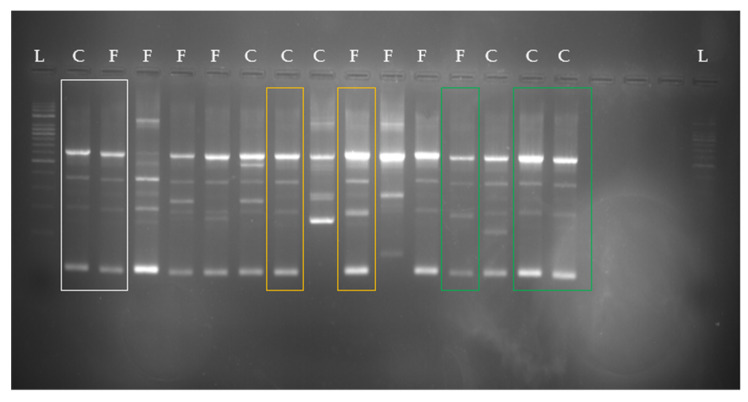
*Staphylococcus sciuri*-RAPD-PCR types from different foster cows and calves. Corresponding pairs are highlighted in the same colours. L = 250 bp ladder; C = calf; F = foster cow.

With respect to *S. scuiri*, several different strains were detected, but in three foster cow–calf pairs, the same strains could be identified. The corresponding foster cow–calf pairs are shown in Figure 4. Isolates from each pair with the same banding pattern are highlighted in the same colour. Moreover, the same strain was detectable in another foster cow from the same group.

For *Sc. suis*, in one foster cow and one of her associated calves, the same strain occurred as in another foster cow from the same group.

### 3.5. Pathogen Matches between Dam and Calf

**Figure 5:**
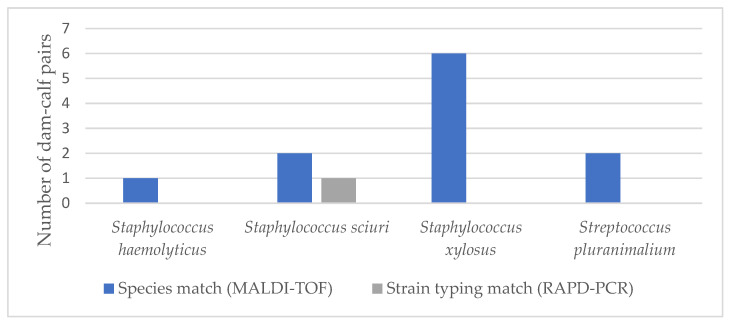
Number of dam–calf pairs and matching pathogens after examination with MALDI-TOF and RAPD-PCR.

In Figure 5, the matching pathogens between the quarter milk samples of the biological dams and the oral swabs of their calves after examination with MALDI-TOF (species match) and RAPD-PCR (strain typing match) are shown. The corresponding table is presented in the supplementary material (Table S3). The same strain of *S. sciuri* was detectable in one cow and her calf. 

## 4. Discussion

The present study aimed to investigate the transmission of mastitis-associated pathogens between foster cows and foster calves during suckling and to evaluate the corresponding consequences for udder health, calf health and internal biosecurity in a foster cow system.

Bacteriological examination was the first step in laboratory analyses of both quarter milk samples and oral cavity swabs. As most of the oral swabs showed a variety of different pathogens growing on blood agar, the examinations focused on the pathogens detected in the quarter milk samples of the associated foster cow and the dam, if present. This should be considered when interpreting the results. Moreover, a certain number of pathogens isolated from the oral swabs could not be identified in the MALDI-TOF, which may have also influenced the results. RAPD-PCR was used to examine isolates from cows and calves for strain similarity. The isolates were first amplified by PCR and then electrophoretically separated according to size. Short primers bound to many different sites on the DNA [22]. This method is considered suitable for strain comparisons in routine diagnostics [27].

### 4.1. Pathogens Detected in the Oral Cavities of Calves

The pathogens identified in the oral swabs of the foster calves were mainly various species of NaS and streptococci, and to a lesser extent, *S. aureus*, *P. multocida* and *T. pyogenes*. NaS constituted the most frequently detected pathogen group; *S. xylosus*, *S. haemolyticus* and *S. sciuri* were the predominating species. So far, there is little literature available regarding the prevalence of NaS in the oral cavities of calves. In previous studies, *S. xylosus* and *S. sciuri* have been successfully isolated from nares and muzzles of heifers [28,29]. Moreover, all three species have been frequently detected on teat skin and teat apices, and in the environments of cattle—for example, in bedding material, stall air and on slatted flooring [30,31,32,33]. Detection in the oral cavities of clinically healthy calves, therefore, does not appear to be surprising. Among the streptococci, *Sc. pluranimalium* was by far the most frequently detected species. It was first described in 1999 by Devriese et al. [34] in tonsils as well as in the genital tracts of cattle and samples of subclinical mastitis. According to Obiger [35], streptococci are part of the normal microbiota of healthy tonsils. This previous study identified 105 strains of streptococci in the tonsils of 62 cattle, including *Sc. uberis* (*n* = 18), *Sc. dysgalactiae* (*n* = 14) and *Sc. agalactiae* (*n* = 3). Daleel and Frost [36] examined tonsils of 300 healthy slaughtered cattle and 100 calves. Of the 293 identified strains of bacteria, 7.3% were attributed to *Sc. dysgalactiae*, 15.8% to *Sc. uberis*, 6.5% to *T. pyogenes* and 7% to *S. aureus*. *Sc. agalactiae* was not detected. They considered bovine tonsils as a significant reservoir for mastitis-associated pathogens such as *Sc. dysgalactiae* and *Sc. uberis*. In the present study, these pathogens could not be isolated from the oral cavities of foster calves.

### 4.2. Matching Pathogens between Cow and Calves

A major aim of the present study was to determine which pathogens can pass from calves to cows and vice versa during suckling. The first investigations on such transmission of pathogens focused on *Sc. agalactiae*. In 1941, Klein and Kleckner [14] examined oral swabs taken from four calves various times after suckling on cows infected with *Sc. agalactiae*. The pathogen was frequently detected up to eight hours afterwards. In a similar study, Schalm [15] fed calves with milk infected by *Sc. agalactiae*. The pathogen was isolable from oral swabs and from teats of one calf after suckling each other. Persistence in the mammary gland until parturition was suspected, since one heifer excreted *Sc. agalactiae* after calving. On the other hand, it has not yet been conclusively clarified whether gastrointestinal or tonsillar colonisation of calves after ingesting infected milk contributes to a further spread of *Sc. agalactiae* within a herd [37]. However, since *Sc. agalactiae* was not detectable in oral swabs or quarter milk samples in the present study, no conclusion could be drawn in this regard. Further investigations with a larger number of study animals could provide more information.

To date, there is little literature available on the transmission of other pathogens such as *Sc. agalactiae* during suckling, although some authors have expressed concerns. For example, Wagenaar and Langhout [7] fear that suckling prevents reliable closure of the teats and thus allows pathogens to enter the mammary glands.

#### 4.2.1. Results of Strain Typing

In the present study, the same pathogen strains were detected for *P. multocida*, *S. aureus*, *S. sciuri* and *Sc. suis* in both quarter milk samples from foster cows and oral cavity swabs from the associated calves by using RAPD-PCR. In addition, isolates of *S. sciuri* were detected in the quarter milk sample of one dam and in the oral cavity of her calf that were found to be identical in the RAPD-PCR.

The most matches were found for *P. multocida*, which could be assigned to two different strains in RAPD-PCR. *P. multocida* is a common coloniser of the upper respiratory system and only occasionally associated with mastitis. Transmission usually occurs from cow to cow, but lymphogenic or haematogenic spread from the respiratory tract to the mammary glands has been discussed [19,38]. In addition, there is evidence of transmission via suckling calves. In his study, Barnum [16] described an outbreak of *P. multocida* in a low-producing herd of 20 dairy cows under poor hygienic conditions. Each dam nursed her calf for several weeks after calving and transmission during suckling was considered very likely, although isolation of *P. multocida* from the oral and nasal swabs of nursing calves was not possible. However, since only swabs from three calves were examined, the significance was rather low. In a more recent study from Spain, the same strain of *P. multocida* was associated with both pneumonia of calves and mastitis in heifers [39]. The authors examined quarter milk samples and nasopharyngeal swabs taken on a heifer replacement farm. *P. multocida* was identified in eleven isolates by species-specific PCR and further analysed by multi-locus sequence typing (MLST). The isolates derived from six calves with pneumonia and five heifers with clinical mastitis. There was no association with nursing calves, as the animals were housed according to age groups. Nevertheless, transmission via suckling seems likely for that study, not only because of strain similarity, but also because the pathogen was found exclusively in the mammary glands of the foster cows at the end of the suckling period, but not in the first quarter milk sample, nor in those from the dams in the milking herd. At this point, further investigations with a control group are required. Moreover, cows with teat lesions have an increased risk of IMI caused by *P. multocida,* as the pathogen can multiply in them [38]. The foster cows in the current study had a significantly higher number of teats with lesions at the end of the foster period (*p* < 0.001), which were probably caused by the calves’ teeth. Thus, an IMI could also be conceivable as a result of the pathogen adhering in teat lesions after the suckling bout.

Using RAPD-PCR, the same strain of *S. aureus* was detected in one foster cow and two of her associated foster calves and in the quarter milk samples of two other foster cows in the same group. *S. aureus* is defined as a contagious major pathogen and transmission takes place especially during the milking process. The mammary gland is considered as the main reservoir [40]. However, *S. aureus* has been previously detected in tonsils of calves and in the oral cavities of intersucking heifers [36,41]. The detection can probably be attributed to the ingestion of infected milk [36,42]. This could also be feasible in the present study, although it cannot be determined whether the foster cows or the foster calves were infected with the pathogen first.

In three foster cow–calf pairs and in one dam and her calf, the same strain of *S. sciuri* was detectable. Although NaS are the most frequently isolated pathogens from bovine milk, *S. sciuri* is only in rare cases a cause of infection and rather considered as a contamination [29,43,44]. Since no consecutive quarter milk samples were taken within a short time, the latter cannot be excluded [44]. So far, a transmission of *S. sciuri* through suckling calves has not been described. Regarding the abundant prevalence in the environment, especially on the teats [30,33], a coincidental matching of pathogens in milk samples and oral swabs of calves after ingestion from the environment seems to be more likely than actual transmission during suckling. Based on the present results, a definitive statement cannot be made.

For *Sc. suis*, the same strain was detected in one foster cow and her associated calf. Originally, *Sc. suis* was detected in swine, where it is associated with various diseases, such as arthritis, meningitis, pneumonia and septicaemia [45]. Isolation from cattle has been previously reported [46,47]. In the study by Okwumabua et al. [47], *Sc. suis* was considered both as a commensal or opportunistic pathogen on mucosal surfaces (conjunctiva and pharynx) or body fluids (milk), and as a causative agent associated with changes in various organs. Cruz-Colque et al. [48] examined tonsils of 60 cattle between the ages of one month and over one year and commonly detected *Sc. suis*, especially in younger animals. On the other hand, *Sc. suis* is not of major importance as a mastitis pathogen in cattle. Transmission during suckling has not been previously described and cannot be conclusively proven with the help of the present results, especially since there was only one case of strain similarity between foster cows and calves.

#### 4.2.2. Other Mastitis-Associated Pathogens

For other mastitis-associated pathogens such as *Sc. agalactiae*, *Sc. dysgalactiae*, *Sc. uberis* and *T. pyogenes*, no strain similarities between cows and calves could be detected in the present study. Apart from *T. pyogenes*, these pathogens were not isolable in the oral cavities of the foster calves. In this context, it should be considered that 25 foster cows left the group before the end of the suckling period, 21 of them because of mastitis. This might have influenced the results of the present study as the calves no longer suckled on these cows, and the results of their quarter milk samples were therefore excluded. On the other hand, these pathogens may have played a role in the transmission between cow and calf, but this can no longer be determined. Furthermore, when interpreting the results, it should be noted that many calves did not suckle exclusively on their associated foster cow. Sucking on different cows occurs mainly when the biological dam is not in the group [17]. According to Kilgour [18], this could lead to calves acting as vectors and transmitting infections between cows. Additionally, it is therefore, more difficult to trace infection routes, as it is not possible to say exactly which calves suckled on which foster cow.

Other specific microorganisms such as *Mycobacterium avium* ssp. *paratuberculosis* and *Salmonella* Dublin were not examined in the present study and subsequently no statement can be made regarding their transmission by suckling.

### 4.3. Consequences for Udder Health, Calf Health and Internal Biosecurity

The second main focus of the present study was to determine the consequences of a potential transmission of pathogens during suckling for udder health, calf health and internal biosecurity.

In terms of udder health, IMI caused by uncommon mastitis-associated pathogens such as *P. multocida* could pose a particular challenge in a foster cow system. Based on the present results and those of previous studies (e.g. [16,39]), transmission during suckling seems very likely. Moreover, alternating suckling of calves might increase the risk of transmission of IMI between different cows within a group. In this context, Kilgour [18] suggested separating the foster cows by age groups, keeping the younger foster cows with lower disease levels separate from older foster cows. However, this requires a certain amount of work and sufficient space.

So far, there is little knowledge concerning the consequences of calf rearing in a foster cow system for the health of the calves. Beaver et al. [4] could not find sufficient evidence for a detrimental effect of a prolonged cow–calf contact in their review. On the other hand, the husbandry of different aged cattle in a group is considered as a risk for the transmission of infectious diseases [6]. The results of the present study provide evidence for a possible transmission of *P. multocida* and *S. aureus* through suckling. *P. multocida* is a common coloniser of the upper respiratory tract and associated with respiratory disease in situations of immunodeficiency and stress [49]. To avoid this, in addition to sufficient intake of colostrum, the acceptance of foster calves and thus the guarantee of milk intake is of particular importance. With regard to *S. aureus*, some authors feared an increased risk of mastitis after ingestion of infected milk. The results of Abb-Schwedler et al. [50] could not confirm an effect of milk infected with *S. aureus* on the udder health of heifers.

Internal biosecurity focuses on preventing the spread of pathogens on a farm. As the transmission of pathogens between different aged cattle is one of the main reasons for early separation of cows and their calves, this is of particular importance in a foster cow system. Therefore, certain precautions are required. In addition to good, stable hygiene, these should especially include careful observations of the animals and immediate removal of sick animals from the group. Most farms separate the foster cow–calf group from the milking herd. Some authors also question the use of cows with chronic mastitis as foster cows. In the study by Wagenaar and Langhout [7], some farmers preferentially selected cows with a high SCC as foster cows. A high SCC indicates a defensive reaction of the body against pathogens, and thus a possible disease event. Suckling a diseased cow conflicts with calls for more natural calf rearing [7]. The altered milk composition with higher albumin or lower casein content could also affect calf growth [51]. On the other hand, previous studies tend to show a positive effect of suckling on udder health [9,10].

## 5. Conclusions

Based on the present results, it could be clarified that transmission of certain mastitis-associated pathogens via suckling is very likely. This resulted in an increase in IMI, especially due to *P. multocida*. On the other hand, transmission from dam to foster cow with the suckling calf as vector could not be clearly established.

## Figures and Tables

**Table 1:** Pathogens isolated from quarter milk samples of foster cows and dams.

Pathogen	Number (% ^1^) of Isolated Pathogens per Sampling (F1, F2, D1)		
	F1	F2	D1
*Streptococcus uberis*	1 (0.3)	2 (0.5)	4 (0.8)
NaS ^2^	29 (7.5)	24 (6.2)	86 (17.8)
*Staphylococcus aureus*	0 (0)	3 (0.8)	4 (0.8)
*Streptococcus dysgalactiae*	0 (0)	2 (0.5)	0 (0)
*Trueperella pyogenes*	0 (0)	2 (0.5)	1 (0.2)
*Escherichia coli*	8 (2.1)	0 (0)	0 (0)
Coliforms	5 (1.3)	0 (0)	0 (0)
*Klebsiella* spp.	2 (0.5)	1 (0.3)	1 (0.2)
*Bacillus* spp.	3 (0.8)	0 (0)	1 (0.2)
*Corynebacterium* spp.	37 (9.6)	50 (13)	31 (6.4)
*Enterococcus* spp.	4 (1.0)	2 (0.5)	0 (0)
*Pseudomonas* spp.	2 (0.5)	0 (0)	0 (0)
Other Streptococci	3 (0.8)	4 (1.0)	2 (0.4)
Lactic acid bacteria	6 (1.6)	0 (0)	0 (0)
*Pasteurella* spp.	0 (0)	13 (3.4)	0 (0)
Others ^3^	2 (0.5)	7 (1.9)	1 (0.2)
Mixed	19 (4.9)	12 (3.1)	16 (3.3)
Contaminated ^4^	22 (5.7)	38 (9.9)	76 (15.7)
In total	121 (31.4)	122 (31.7)	147 (30.4)
No specific growth ^5^	242 (62.9)	225 (58.4)	260 (53.8)

^1^ Percentage related to all examined quarter milk samples per sampling. ^2^ Non-*aureus* staphylococci. ^3^ *Aerococcus viridans*, *Mannheimia haemolytica*, *Micrococcus* spp. ^4^ More than two different pathogens detected in one sample. ^5^ No bacteriological growth on blood agar.

**Table 2:** Pathogens and pathogen groups identified in oral cavity swabs.

Pathogen/Pathogen Group	*n* (%)	Pathogen Species (*n*)
NaS ^1^	531 (34.3)	*Staphylococcus (S.) xylosus* (195)
		*S. haemolyticus* (107)
		*S. sciuri* (88)
		*S. gallinarum* (48)
		*S. equorum* (32)
		*S. arlettae* (18)
		*S. vitulinus* (18)
		*S. chromogenes* (9)
		*S. auricularis* (5)
		*S. lentus* (4)
		*S. epidermidis* (3)
		*S. succinus* (2)
		*S. cohnii* (1)
		*S. gallinaceus* (1)
Streptococci	418 (27)	*Streptococcus (Sc.) pluranimalium* (271)
		*Sc. suis* (64)
		*Sc. ovis* (16)
		*Sc. orisratti* (12)
		*Sc. henryi* (8)
		*Sc. minor* (7)
		*Sc. gallolyticus* (6)
		*Sc. sanguinis* (6)
		*Sc. gallinaceus* (5)
		*Sc. oralis* (5)
		*Sc. devriesei* (4)
		*Sc. lutetiensis* (3)
		*Sc. hyovaginalis* (2)
		*Sc. thoraltensis* (2)
		*Sc. canis/Sc. dysgalactiae* (1)
		*Sc. gallinarum* (1)
		*Sc. infantarius* (1)
		*Sc. mitis* (1)
		*Sc. mitis/oralis/peroralis* (1)
		*Sc. parasanguinis* (1)
		*Sc. ratti* (1)
*Corynebacterium* spp.	137 (8.9)	*Corynebacterium (C.) xerosis* (97)
		*C. stationis* (9)
		*C. glutamicum* (8)
		*C. camporealensis* (5)
		*C. freneyi* (4)
		*C. casei* (3)
		*C. propinquum* (3)
		*C. callunae* (2)
		*C. efficiens* (2)
		*C. amycolatum* (1)
		*C. flavescens* (1)
		*C. stimulans* (1)
		*C. urealyticum* (1)
*Aerococcus viridans*	93 (6)	
*Rothia nasimurium*	66 (4.3)	
*Pasteurella multocida*	49 (3.2)	
*Staphylococcus aureus*	41 (2.6)	
*Bacillus* spp.	40 (2.6)	*Bacillus (B.) pumilus* (16)
		*B. altitudinis* (8)
		*B. subtilis* (7)
		*B. licheniformis* (4)
		*B. clausii* (3)
		*B. subtilis* group (2)
*Acinetobacter* spp.	28 (1.8)	*Acinetobacter (A.) johnsonii* (9)
		*A. pittii* (9)
		*A. schindleri* (4)
		*A. lwoffii* (2)
		*A.* spp. (2)
		*A. indicus* (1)
		Member of *A. baumnnii/calcoaceticus* complex (1)
*Candida albicans*	20 (1.3)	
*Enterococcus* spp.	17 (1.1)	*Enterococcus (E.) faecium* (12)
		*E. casseliflavus* (3)
		*E. aquimarinus* (1)
		*E. villorum* (1)
*Mannheima* spp.	16 (1)	*Mannheimia (M.) haemolytica* (10)
		*M. varigena* (6)
*Pseudomonas* spp.	11 (0.7)	*Pseudomonas (P.) monteilii* (4)
		Member of *P. stutzeri* group (2)
		*P. aeruginosa* (1)
		*P. extremorientalis* (1)
		*P. fulva* (1)
		*P. stutzeri* (1)
		*P. synxantha* (1)
*Trueperella pyogenes*	10 (0.6)	
*Micrococcus* spp.	9 (0.6)	*Micrococcus (M.) luteus* (3)
		*M. lylae* (6)
*Pantoea agglomerans*	8 (0.5)	
*Paenibacillus* spp.	7 (0.5)	*Paenibacillus (P.) lactis* (3)
		*P.* spp. (3)
		*P. woosongensis* (1)
*Escherichia coli*	5 (0.3)	
*Stenotrophomonas* spp.	5 (0.3)	*Stenotrophomonas (S.) maltophilia* (3)
		Member of *S. maltophilia* (1)
		*S.* spp. (1)
Others	37 (2.4)	*Brevibacterium luteolum* (1)
		*Cellulosimicrobium cellulans* (1)
		Synonym of *Cryptococcus neoformans* (1)
		*Curtobacterium flaccumfaciens* (1)
		*Desemezia incerta* (2)
		*Enterobacter cloacae* (3)
		*Erwinia persicina* (1)
		*Exiguobacterium* spp. (1)
		*Glumaticibacter arilaitensis* (1)
		*Granulicatella adiacens* (1)
		*Helococcus ovis* (1)
		*Klebsiella oxytoca* (1)
		*Kocuria carniphila* (4)
		*Macrococcus canis* (2)
		*Microbacterium* spp. (4)
		*Moraxella boviculi* (3)
		*Neisseria* spp. (3)
		*Ochrobacterium intermedium* (1)
		*Oerskovia tubata* (1)
		*Psychrobacter* spp. (1)
		*Serratia marcesens* (1)
		*Solibacillus silvestris* (1)
		*Weissella hellenica* (1)
In total	1548 (100)	

^1^ Non-*aureus* staphylococci.

## Data Availability

All existed data are listed in the manuscript.

## Supplementary Materials

The following are available online at https://www.mdpi.com/article/10.3390/ani11092738/s1. Table S1: Primers used for RAPD-PCR. Table S2: Matched pathogens between foster cows and associated calves after examination with MALDI-TOF and RAPD-PCR. Table S3: Matched pathogens between dams and calves after examination with MALDI-TOF and RAPD-PCR.
