# Supplementary material for: Investigations on Transfer of Pathogens between Foster Cows and Calves during the Suckling Period
Source: Animals (Basel). 2021 Sep 19;11(9):2738. doi: 10.3390/ani11092738 (PMC8469241; doi:10.3390/ani11092738)
Supplement: Supplementary file 1 [file animals-11-02738-s001.zip › animals-1373986-supplementary.pdf]

animals

Supplementary Materials

Investigations on Transfer of Pathogens between Foster Cows and Calves during the Suckling Period

Katharina Köllmann [1], Nicole Wente [1], Yanchao Zhang [1] and Volker Krömker [2,*]

[1] Department of Microbiology, Faculty of Mechanical and Bioprocess Engineering, University of Applied Sciences and Arts, 30453 Hannover, Germany; katharina.marie.koellmann@tiho-hannover.de (K.K.); nicole.wente@hs-hannover.de (N.W.); yanchao.zhang@hs-hannover.de (Y.Z.)
[2] Department of Veterinary and Animal Sciences, University of Copenhagen, 1870 Frederiksberg C, Denmark
* Correspondence: Volker.Kroemker@sund.ku.dk

Citation: Köllmann, K.; Wente, N.; Zhang, Y., Krömker, V. Investigations on Transfer of Pathogens between Foster Cows and Calves during the Suckling Period. *Animals* **2021**, *11*, 2738. https://doi.org/10.3390/ani11092738

Academic Editor: Ylva Persson and Mizanur Rahman

Received: 25 August 2021
Accepted: 15 September 2021
Published: 19 September 2021

Publisher's Note: MDPI stays neutral with regard to jurisdictional claims in published maps and institutional affiliations.

Copyright: © 2021 by the authors. Submitted for possible open access publication under the terms and conditions of the Creative Commons Attribution (CC BY) license (https://creativecommons.org/licenses/by/4.0/).

1. Further Details of the Farm

The present study was conducted on a large farm in Eastern Germany, which is managed in accordance with European and German organic standards. The herd comprises a total of 1500 Holstein Friesian cows and their offspring. Dairy cows are kept in different groups according to their milk yield in a cubicle housing system with curtains for extreme weather conditions. The bedding material consists of a mixture made from spelt husks, straw, lime and water. All cows have access to pasture from April (May) to October. Milking is done twice a day in a rotary milking parlour.

The foster cows and their associated calves are kept apart from the milking herd in a separate housing system with the dry cows. The calving area is also located in this barn. After calving, the cows are usually milked immediately and each calf receives at least 3 litres of colostrum from a bucket, preferably that of the dam. If the dam does not have enough milk or the colostrum quality is insufficient, stored colostrum is used. Each cow and her calf/ calves spend the following 3-5 days together. During this time, the cows suckle their calves and are additionally milked twice a day on a pipeline milking system. The selection of foster cows and the subsequent habituation phase are described in the article. The group of 16 foster cows and associated calves stays together until weaning. Both female and male calves were reared by foster cows. The area for foster cows and calves consists of a feeding area with slatted flooring and a resting area made of straw. Adjacent to the latter is an area that is only accessible to calves. There, calf cereal, hay and water are available to them. The udder, teats and milk appearance of the foster cows are checked daily by the staff. If treatment is necessary, the foster cows are removed from the group. Since not all sick foster cows can be replaced, each foster cow is usually initially assigned three calves. In this way, each foster cow has a free teat as a reserve, so to speak. Gradual weaning of foster calves begins at an age of approximately three months. During weaning, the calves are initially locked away from the foster cows for hours at time. Duration of separation is progressively prolonged. After weaning, the foster cows return to the milking herd and are kept in a group with freshly calved cows.

Regarding the udder health situation, all relevant mastitis pathogens such as *Staphylococcus. aureus* or *Streptococcus. agalactiae* are prevalent in the herd. During summer 2020, problems occurred with intramammary infections due to *Klebsiella* spp. and led to the loss of multiple cows. With respect to foster cows, clinical mastitis caused by *Pasteurella* spp. was described as a common problem. The farm is considered free from *Mycobacterium avium* ssp. paratuberculosis.

2. Strain Comparisons

The primers used for Randomly Amplified Polymorphic DNA-Polymerase Chain Reaction (RAPD-PCR; [1] and their corresponding PCR temperature profiles are shown in Table S1.

Table S1. Primers used for RAPD-PCR.

Primer	Pathogens	Temperature Profile
ERIC-1R (5′-ATGTAAGCTCCTGGGGAT-TCAC-3′) [2]	*Staphylococcus aureus*<br>*Staphylococcus arlettae*<br>*Staphylococcus sciuri*<br>*Trueperella pyogenes*<br>*Escherichia coli*	1 × 94 °C/2 min<br><br>35 × 94 °C/1 min<br>25 °C/1 min<br>72 °C/4 min<br><br>1 × 94 °C/1 min<br>25 °C/1 min<br>72 °C/8 min
OPE-04 (5′-GTGACATGC C-3′) [3]	*Aerococcus viridans*<br>*Acinetobacter lwofii*<br>*Corynebacterium xerosis*<br>*Streptococcus pluranimalium*<br>*Streptococcus suis*<br>*Pasteurella multocida*	1 × 95 °C/2 min<br><br>35 × 80 °C/0.5 min<br>70 °C/0.5 min<br>60 °C/0.5 min<br>50 °C/0.5 min<br>40 °C/0.5 min<br>33 °C/1 min<br>72 °C/2.17 min
C RAPD Staph (5′- CGGGGGACTGTT-GGGCGCCATCT-3′) [4]	*Staphylococcus xylosus*<br>*Staphylococcus sciuri*	1 × 95 °C/1 min<br><br>45 × 95 °C/0.5 min<br>45 °C/1 min<br>75 °C/2 min<br><br>1 × 72 °C/1 min
Primer A (5′-CTGGCGGCTTG-3′) [5]	*Staphylococcus haemolyticus*	1 × 95 °C/3 min<br><br>45 × 95 °C/0.25 min<br>34 °C/1 min<br>72 °C/2 min<br><br>1 × 72 °C/10 min

3. Pathogen Matches between Cows and Calves

Table S2 shows the matched pathogens between foster cows and calves after examination with MALDI-TOF (species match) and RAPD-PCR (strain typing match).

Table S2. Matched pathogens between foster cows and associated calves after examination with MALDI-TOF and RAPD-PCR.

Pathogens	Species Match (MALDI-TOF)	Strain Typing Match (RAPD-PCR)
Acinetobacter lwoffi	1	0
Aerococcus viridans	6	0
Corynebacterium xerosis	1	0
Escherichia coli	2	0
Pasteurella multocida	9	8 [1]
Staphylococcus arlettae	1	0
Staphylococcus aureus	2	2

Staphylococcus haemolyticus	18	0
Staphylococcus sciuri	5	3
Staphylococcus xylosus	2	0
Streptococcus pluranimalium	13	0
Streptococcus suis	2	1
Trueperella pyogenes	1	0

[1] One isolate failed to grow again after storage.

The number of foster cow–calf pairs in which the same pathogen was found both in the quarter milk sample of the foster cow and the oral swab of the associated foster calf is indicated. The second column of the table refers to the results of the MALDI-TOF, the third to those of the RAPD-PCR. For instance, *Acinetobacter lwoffii* was detectable in the quarter milk sample of a foster cow as well as in the oral cavity of one of her associated calves. Based on chromosome banding, the two isolates showed no match in the subsequent RAPD-PCR.

Table S3 shows the matching pathogens between the quarter milk sample of the biological dams and the oral swab of their calf after examination with MALDI-TOF (species match) and RAPD-PCR (strain typing match). The same strain of *S. sciuri* was detectable in one cow and her calf.

Table S3. Matched pathogens between dams and calves after examination with MALDI-TOF and RAPD-PCR.

Pathogens	Species Match (MALDI-TOF)	Strain Typing Match (RAPD-PCR)
Staphylococcus haemolyticus	1	0
Staphylococcus sciuri	2	1
Staphylococcus xylosus	6	0
Streptococcus pluranimalium	2	0

4. References

1. Williams, J.G.K.; Kubelik, A.R.; Livak, K.J.; Rafalski, J.A.; Tingey, S.V. DNA polymorphisms amplified by arbitrary primers are useful as genetic markers. *Nucleic Acid Res.* **1990**, *18 (22)*, 6531–6535. 10.1093/nar/18.22.6531
2. Vogel, L.; Jones, G.; Triep, S.; Koek, A.; Dijkshoorn, L. RAPD typing of *Klebsiella pneumoniae, Klebsiella oxytoca, Serratia marcescens* and *Pseudomonas aeruginosa* isolates using standardized reagents. *Clin. Microbiol. Infect.* **1999**, *5 (5)*, 270–276. 10.1111/j.1469-0691.1999.tb00140.x
3. Gillespie, B.E.; Jayarao, B.M. Pankey, J.W.; Oliver, S.P. Subtyping of Streptococcus dysgalactiae and Streptococcus uberis isolated from bovine mammary secretions by DNA fingerprinting. *J. Vet. Med.* **1998**, *45*, 585–593. 10.1111/j. 1439–0450.1998.tb00831.x
4. Daminani, G.; Telecco, S.; Comincini, S.; Sironi, M.; Carretto, E.; Marone, P. Comparison of an improved RAPD fingerprinting with different typing methods for discriminating clinical isolates of *Staphylococcus* spp. *Eur. J. Cardiovasc. Prev. Rehabil.* **1996**, *12*, 163–169. 10.1007/BF00145502
5. Hijazin, M., Sammra, O., Ulbegi-Mohyla, H., Nagib, S., Alber, J., Lämmler, C., Kämpfer, P., Glaeser, S.P., Busse, H.J., Kassmannhuber, J., Prenger-Berninghoff, E., Weiss, R., Siebert, U., Hassan, A.A., Abdulmawjood, A., Zschöck, M. *Arcanobacterium phocisimile* sp. nov., isolated from harbour seals. *Int. J. Syst. Evol. Microbiol.* **2013**, *63*, 2019–2024. 10.1099/ijs.0.045591–0
